# “We Are Here for You All the Way”—Patients’ and Relatives’ Experiences of Receiving Advanced Home Care

**DOI:** 10.1177/1049909120905259

**Published:** 2020-02-13

**Authors:** Ingela Wennman, Anna Ringheim, Helle Wijk

**Affiliations:** 1 Department of Strategic Planning, Sahlgrenska University Hospital, Göteborg, Sweden; 2 Institute of Caring Sciences and Health, Sahlgrenska Academy, Gothenburg University, Göteborg, Sweden; 3 Department of Quality Development, Sahlgrenska University Hospital, Göteborg, Sweden; 4 Gothenburg Center of Person Centred Care (GPCC), Gothenburg University, Göteborg, Sweden; 5 Center for Health Care Architecture, Chalmers University, Göteborg, Sweden

**Keywords:** palliative care, quality of care, advanced home care

## Abstract

**Background:**

It is of great importance to understand how patients and their close relatives experience the pros and cons of advanced home care so as to further develop this quickly growing choice of care.

**Objective:**

The aim of this study was to explore the experiences of receiving advanced home care among patients affected by life-threatening illness and their close relatives.

**Design:**

The study was an interview study conducted with patients in their homes. Some patient interviews were conducted together with a close relative participating.

**Setting/Participants:**

Patients registered in advanced home care in 2017 were offered the opportunity to participate in the study. The selection criteria were that the patient was within grade 3 of the Eastern Cooperative Oncology Group’s Performance Status, older than 18 years, able to orient to time and place, and not newly registered.

**Analysis:**

The interviews were recorded and transcribed verbatim and analyzed with qualitative content analysis.

**Results:**

A total of 11 interviews were conducted: 8 with patients and 1 or 2 close relatives together; and 3 with the patient alone. It resulted in 3 main categories: create a safe environment, see the person, and better to manage care at home.

**Conclusion:**

The results of this study show that patients and close relatives perceived that advanced home care was a safe and secure form of caring during advanced as well as end-of-life care.

## Background

The lack of consensus regarding concepts and definitions for palliative care in general hinders the provision of good and equitable care worldwide, according to the World Health Organization (WHO).^1^ In general, this is also true for Sweden, as highlighted by the National Board of Health and Welfare.^2^ In this study, we therefore explored the experiences of receiving advanced home care among palliative patients with various diagnosis and their close relatives. The rationale for choosing the special setting of advanced home care for this study is that it is characterized by clearly defined concepts and clearly formulated goals and guidelines, in contrast to the situation in palliative care in general.

Despite widespread acceptance of the WHO definition of palliative care,^3^ the lack of an international agreement stating the requirements of performing good quality of care in the final stages of life remains a challenge. Of the 95% of patients within palliative care most have had cancer diagnosis. To reduce the risk of inequality toward patients with other diagnosis in need of palliative care, the European Association for Palliative Care (EAPC) recommends the implementation of quality standards, including a multidisciplinary palliative team with specialist competence within palliative care, to offer advanced home care to all patients in need of palliative care despite diagnosis and to support close relatives.^4^

Approximately 100 000 people die in Sweden every year, many of them in need of palliative care.^5^ The National Board of Health and Welfare requires that palliative care should be based on teamwork, and the Swedish national guidelines for palliative care highlight the importance of symptom management, communication, building up good relationships, and providing support to close relatives,^5^ which is in line with the EAPC recommendations. However, despite this, palliative care provision is still performed and distributed equally throughout the country.^6^

Advanced home care is a specialized form of palliative care performed in Sweden, which is characterized by multiprofessional teams of specialists who have the commission of caring for severely ill people with various diagnosis in their home and relieving their suffering for varying periods of time.^7^ This policy aligns with international developments in palliative care, where home care is becoming more common in many parts of the world.^4^ Advanced home care presents an opportunity for the specialist team to meet the needs of patients and their close relatives with a person-centered approach and to offer individual solutions and shared decision-making.^5^ In summary, it can be assumed that an advanced home-care model as advanced home care generates a high level of quality of care and quality of life for patients and their close relatives. It is therefore important for the further development and improvement of palliative care in general to explore how patients and their close relatives in advanced home care experience the pros and cons of this care.

## Objective

The aim of this study was to explore patients in need of palliative care despite diagnosis, and their relatives’ experiences receiving advanced home care.

## Design

The study was an exploratory, qualitative study which was conducted with patients alone or with the patient together with a close relative in their homes. The authors used data from unstructured in-depth interviews that were recorded and transcribed for analysis purposes. The rationale for using this interview format was to create an open atmosphere and simulate patients and relatives to speak out freely about their experiences. The interviews started with a general open-ended question: “What does advanced home care mean to you?” followed by supplementary questions to deepen the understanding of the participants’ experiences. The supplementary questions targeted areas concerning experiences of participation in the care provided, pros and cons achieving care in a private home, and aspects importing for the experience of the save and secure care. The interviews were conducted by 4 researchers who were not involved in the care. The researchers worked in pairs. The reason for using this approach was to support the ambition of creating an open atmosphere and to stimulate patients and relatives to speak out freely about their experiences. By being 2 researchers instead of 1 made the interview situation being more of a conversational style than a regular interview. The patients were informed and agreed to this approach in advance.

## Setting/Participants

In Gothenburg, advanced home care was implemented in 2011. Around 200 patients are continuously registered in the system at any one time, most of them having had a cancer diagnosis in a palliative stage. The care is provided in the patients’ home in cooperation between municipal and inpatient care, provided 24 hours per day, 7 days per week, to patients older than 18 years.

The recruitment strategy followed a strict process: The advanced home care teams identified potential patients and relatives, registered in advanced home care in March/April 2017, who were invited to voluntary participate in the study. The main selection criterion was based on grade 3 of the Eastern Cooperative Oncology Group’s (ECOG) Performance Status,^8^ which means that the patient is bedridden for less than 50% of the day. In addition, patient participants were older than 18 years, could orient to time and space, and were not newly registered. They were also selected where the burden of an interview was not considered to be too heavy. To assure that, the patients and relatives were contacted again in close connection with the interview to confirm that they still wanted to participate in the interview and that the time selected was appropriate. A total of 11 interviews were conducted: 8 with patients and 1 or 2 close relatives together, and 3 with the patient alone. Of the patients, 8 were men and 3 were women, all between the ages of 53 and 87 years. The close relatives were either their partner or an adult child and, in one interview, a sister-in-law. Three patients did not have a Swedish background but were fluent in Swedish. The length of the interviews varied due to the serious conditions of the patients and lasted between 17 and 70 minutes (see Table 1).

**Table 1.** Participants in the Interviews.

Interviews	Patient	Age	Close Relative
1	Man	61	Wife
2	Women	59	Husband
3	Man	75	-
4	Woman	53	-
5	Man	72	Sister-in-law
6	Man	77	Wife
7	Man	58	-
8	Man	54	Wife
9	Man	77	Sambo
10	Women	87	Daughter
11	Man	65	Wife
Sum	11	-	8

## Analysis

The interviews were recorded and transcribed verbatim and analyzed with qualitative content analysis.^9,10^ All interviews were read through by the research team to get an overall sense of content. Meaning-bearing units were then identified in relation to the aim of the study. The researchers worked in pairs to condense meaning-bearing units, code and categorize the essence of the material, and then bring them together in themes. The content of the themes was illustrated by citations drawn from the interviews. Between steps, the research team convened for review and to reach consensus regarding meaning-bearing units, coding, categorization, and themes.

## Ethical Considerations

Patients and relatives who receive advanced medical care at home belong to a vulnerable group and are in a dependent position in relation to health care. Interviews about the performance and content of care can cause anxiety and emotions that lead to discomfort. It is therefore important that the researchers are responsive to such signals in the interview situation. Before the interviews began, participants were given the background to the study and were told how the results were to be used. It was reiterated that their participation was entirely voluntary and that they could withdraw at any time without providing any reasons. The participants were also invited to review the final results both by reading the final report and attending a follow-up meeting discussing the result. The study was conducted according to the ethical principles of the Declaration of Helsinki.^11^ The study was approved by the Regional Ethical Review Board in Gothenburg (Dnr; 1000-16).

## Results

The analysis of the interviews resulted in 3 themes: create a safe environment, see the person, and better to manage care at home. The 3 themes comprise of a variation of aspects of the theme illustrated by quotations from the interviews.

### Create a Safe Environment

The need for safety was significant and was mentioned by all patients and close relatives. For example, patient and relatives described how the team responded both to explicit and unspoken significant needs, such as pain relief, communication with health-care professionals, and psychosocial support. This was achieved by the staff reassuring patients and close relatives that they could call the care service around the clock, and having staff call regularly to inquire about how the patient was feeling and to determine whether any efforts were needed to meet their needs.

Many mentioned in positive terms that the transition from curable to palliative care had become transparent by involving them in discussions about the decision-making process. However, some experienced that the transition to advanced home care went too quickly and that they would prefer to “slow down” the process. For example, some experienced the conversation concerning the transition to advanced home care as being characterized by having received to compact information, which could be perceived as frightening. Also, since there were many actors involved in this transition, for example, physicians from the hospital, nurses from the municipality, and physiotherapists from primary care, the patients and relatives expressed an uncertainty about who was responsible for the different caring actions. However, others felt that the holistic overall approach, significant in palliative care, created a positive difference. It meant that all their needs were addressed, and that the written information provided them with the opportunity to return to different parts and also to find appropriate contact details if there were any questions.

Many patients mentioned that it was remarkable that the various health-care providers used different documentation systems for the patient records, which they felt had a negative impact on safety. One patient described how the staff used sticky notes to communicate with other health-care providers. Another patient experienced that cooperation could be improved between advanced home care and the attending physician at the hospital.

They can’t see the notes…it’s probably wrong, because they can’t see the patient records…unable to solve it with any consultant—or log in, so it’s a pure stone-aged action to sit and send a test result and other stuff by fax. So that’s too bad.

Some relatives stressed the importance of not having to bear too much responsibility for making decisions about treatments and possible relocation to the hospital. It was especially crucial for those relatives who worked to be assured that the care environment was safe, so that they might dare to go to work and not have to stay home. Alternatively, some family members highlighted the importance of participation in terms of balancing the responsibility of the advanced home care team and the close relatives. One participant expressed this as a need for increased involvement in pain management medications, for example, to be enabled to provide rapid pain relief. The competence of the staff, adopting a holistic view and pedagogical ability, was emphasized, both regarding information of a medical nature and about the available support systems. It was important to obtain clear information about who to contact for specific nursing measures when health needs increased and when the patient’s ability to take care of themselves decreased.

### See the Person

All patients and close relatives raised the importance of seeing the patient as a person, not just “a patient.” They expressed this by describing how they felt pampered by the staff when they were being supportive, empathetic, and helpful.

And I actually discussed the matter with the doctor here, we sat down and talked about it for almost an hour, about how things would turn out and how it (dying) would be. She said; “We are here for you all the way.” “All the way,” now I know exactly what they mean.

The quality of the relationship was an important factor in the encounters between staff, patients, and close relatives. Some patients mentioned that a precondition for a good relationship was continuity among the staff, which in turn contributed to a relaxed and personal relationship. Others thought this was less important and could see an advantage in the opportunity to meet different carers, which they described as stimulating. However, the importance of having a permanent care coordinator who could take responsibility for continuity and special needs throughout the care trajectory was crucial by many. Both patients and relatives stressed the importance of that the staff safe guarded the patients personal integrity, for example, in terms of keeping to agreed visiting times and disclosing whether anything had prompted the visit. Receiving advanced care 24/7 in the home could also be connected with a sense of helplessness, as the disease affected the patients’ ability to perform daily activities. This created increased dependency in relation to both health-care providers and relatives and a need for various technical aids and medical equipment. Some patients described this helplessness as a feeling of being discounted, for example, because the staff needed to have access to keys to be able to visit them around the clock. Conversely, some patients said that being aware that the staff had a key contributed to reducing these feelings of helplessness and reassured them that help could be obtained quickly. The team’s ability to adapt the visits to meet the patients’ needs was highly appreciated and engendered the feeling that nothing was impossible. The patients described how the advanced home care team could adapt their service at short notice if needed and also how they were willing to test individual solutions when problems emerged. This created a sense of calmness. However, this was not always the case. The patients described that since their well-being fluctuate during the day and that the energy needed to conduct various forms of activities therefore could easily change on a daily basis which sometimes caused problems because it was not possible for the complex health-care system to respond to these rapid changes. For example, a patient described how she was not able to visit her summerhouse in the moment when she had the energy for it, as it was situated in another county, which required extensive planning for the staff and organization.

### Better to Manage Care at Home

Both patients and close relatives appreciated that being a patient in advanced home care did not necessitate visits to the hospital via the emergency department (ED). This was described as being a major relief. They described that visiting the ED was characterized by uncertainty, as the staff did not know the medical history, there were long waiting times and a stressful environment, which, together, placed great strain on them. They also reflected on different scenarios before advanced home care was implemented and recalled a period of distress in not having a personal contact to turn to.

I only get reminded of a lot of negative things by visiting the hospital. For me it’s just, if I go to the hospital I have to recover during the following 24 hours, it is nothing but negative…it’s much better if everything could be managed here (at home).

Being offered support resources at home, such as treatments and technical aids, was important, as were existential conversations. This also meant that advanced home care took responsibility for contacting other stakeholders in the transition of care, for example, when applying for residency at the hospice. Easy accessibility to the services available within advanced home care created a safe environment and provided effective support for both patients and their close relatives, a support that the health-care providers were not able to achieve in ordinary acute care.

## Discussion

The results show that, overall, patients despite diagnosis and their close relatives were satisfied with the quality of advanced home care and expressed that they wished that they would have been offered advanced home care earlier. Also revealed was that home health care was experienced as a safe form of care with the team establishing a close relationship with the patient. These findings align with studies conducted within other specialties, but which have the same ambition of implementing a person-centered approach to accomplish safe care of high quality.^12,13^ The short waiting time and individual solutions that characterize advanced home care were interpreted as contributing to safety among the patients and their close relatives especially when comparing their previous experiences of emergency care, which featured long waiting times, contributing to anxiety and suffering. This finding is confirmed by other studies, which stress the relationship between long waiting times and negative experiences of care^14,15^ and the importance of being acknowledged and involved in care decisions and to feel worthy of having a place at the ED.^16^ This is in line with findings from Gustavsson,^17^ illuminating the importance of viewing care from a patient perspective, which may help overcome existing gaps in the organizational structure, such as separation into specialist functions. However, Gustavsson^17^ also presents challenges in line with our experiences such as that patient involvement places patients and health-care professionals in new roles as codesigners, which calls into question prevailing roles and relationships. Sometimes the organizational system clashed with the person-centered approach, for example, when staff from the various stakeholders, who held other roles and were bound by different regulations, were involved in the care or when IT systems were not compatible. This sometimes made it difficult to resolve individual issues for the patient and their close relatives due to the many actors involved, together with the acute condition of the patients, which often could change from 1 hour to the next. Not having to seek emergency care when the patient’s condition suddenly changed was appreciated most. The stressful atmosphere at the ED, together with the long waiting times there, was described as being challenging, both physically and psychologically. Due to the patient’s condition, with symptoms of severe pain and fatigue, the patient’s vulnerability is increased when sustaining the huge effort involved in making a visit to the ED,^18^ which can lead to increased risk of morbidity and death.^19^

Although there were many patients registered in advanced home care, it was difficult to find patients who were strong enough, despite their palliative stage, to participate in the interviews, and, therefore, the recruitment of participants took longer than expected. Some patients agreed to participate but had to decline due to a deterioration in their disease status. Others chose to attend the interview despite their condition having worsened between their agreement to join the study and the time of the interview, which could be perceived as an ethical dilemma, as the agreed inclusion criterion that was based on grade 3 of ECOG’s Performance Status^8^ 3 had to be reconsidered. The participants were invited to take part of the results by reading the final report and to discuss its findings on a follow-up meeting. This was greatly appreciated by all participants who took advantage of reading the report in addition some of the relatives also attended a follow-up meeting.

### Strengths and Limitations

We consider that it is a strength that the researchers were not part of providing the usual care for the participants and thereby did not have an existing relationship with them. The fact that the interviews always were performed with 2 researchers attending could be questioned. There is a risk of an unbalance during the interview making the patient experiencing an underdog situation. On the other hand by being 2 researchers made it possible to support a conversational environment and a comfortable situation. In addition, conducting the interviews at the patient’s home somewhat reduced the researcher–participant hierarchy and made it possible for the interviewers to be humble and to adapt appropriately to the situation, as well as to support meaningful participation. The open questions also allowed the participants to speak freely about their experiences of advanced home care, which in turn provided rich material. In addition, the material does not include separate interviews with close relatives, which limits the possibility of obtaining more profound knowledge of their particular experiences. The result is presented in 3 themes which can be regarded as a limitation since it is not strictly following the methodology of content analysis. However, the reason for not dividing the content of the themes into additional subthemes was to avoid the risk of fragment the content.

### Future Studies

From our findings, it was evident that providing safe advanced home care of high quality is a most prominent question, not only for the patients themselves but also for their close relatives. Because the interviews in this study focused mainly on the patients’ experiences of being provided palliative care at home, aspects that are focused on the experiences of being a close relatives and carer for patients enrolled in advanced home care must also be further explored. In addition, our target group was restricted to only inviting adults/older persons. Therefore, a wider age range of participants should be included in our coming studies.

## Conclusions

The results of this study show that patients and close relatives estimate that advanced home care is a safe form of care. Furthermore, the results show that there is potential for expanding the enrollment of patients, which many emphasized. The enrollment process was perceived by many to provide too much information at once and was sometimes difficult to understand in terms of its content, roles, and responsibilities. Patients and relatives expressed their wish to be more involved, both in the meetings and in the planning. Providing advanced health care at home is a relatively new form of care with little empirical evidence on its effectiveness to date. The main body of research concerns in-patient palliative care or care provided at hospices which is not similar to advanced home care care. The experience of those involved in the provision and receipt of advanced care at home should therefore be explored further.
